# Research and progress of cGAS/STING/NLRP3 signaling pathway: a mini review

**DOI:** 10.3389/fimmu.2025.1594133

**Published:** 2025-05-20

**Authors:** Ke-qian Chen, Wen-rui Tang, Xiang Liu

**Affiliations:** ^1^ Department of Clinical Pharmacy, Xiangtan Central Hospital (The Affiliated Hospital of Hunan University), Xiangtan, China; ^2^ Department of Infection Control, Xiangtan Central Hospital (The Affiliated Hospital of Hunan University), Xiangtan, China

**Keywords:** cGAS, STING, inflammasome, NLRP3, IFN

## Abstract

Over the past decade, inflammasome and cGAS-STING have received significant attention as immune components that coordinate host immune homeostasis. Although the inflammasome and cGAS-STING are relatively independent immune signaling pathways, some studies have pointed out that there is an important association between cGAS-STING and the inflammasome. As the downstream of cGAS-STING, inflammasome plays an important role in many diseases. In this mini-review, we review the association between CGAS-STING and NLRP3 inflammasome, the progression of the cGAS/STING/NLRP3 signaling pathway in the pathogenesis of disease, the therapeutic strategies that targeted cGAS/STING/NLRP3 signaling pathway. We believe that targeting the cGAS/STING/NLRP3 signaling pathway in the future has broad prospects and significance for the treatment of disease.

## 1 Introduction

In 2008, American scientist Ishikawa H and his team found a transmembrane protein “Stimulator of Interferon Genes (STING)” (1). STING, also known as TMEM173, MITA, ERIS, and MPYS, is composed of 379 amino acids and has a molecular weight of 42 kU (2). STING is widely distributed in the endoplasmic reticulum of mammalian immune cells. Meanwhile, STING plays an important role in regulating the innate immune response. Activation of STING confers host immunity and is key to clearing a wide range of pathogens (viruses and bacteria), while its absence causes cells to fail to respond to cytoplasmic DNA or certain bacterial components (3). Subsequently, in 2013, Sun L and his team discovered that cytoplasmic DNA catalyzes the production of cGAMP by activating cyclic GMP-AMP synthase (cGAS) (4). cGAS, also known as C6ORF150 or MB21D1, is composed of 522 amino acids and has a molecular weight of 60 kU (5). As an innate immune sensor for cytoplasmic double-stranded DNA (dsDNA), the C-terminal of cGAS contains a nucleotide transferase domain for catalysis and multiple DNA binding sites (6). The inflammasome is a multiprotein complex in the cytoplasm. Meanwhile, it is also a relatively important part of the immune system. Over the past decade, inflammasome and cGAS-STING have received significant attention as immune components that coordinate host immune homeostasis. Although the inflammasome and cGAS-STING are relatively independent immune signaling pathways, some studies have pointed out that there is an important association between cGAS-STING and the inflammasome. As the downstream of cGAS-STING, inflammasome plays an important role in many diseases. In this mini-review, we review the association between cGAS-STING and NLRP3 inflammasome, the progression of the cGAS/STING/NLRP3 signaling pathway in the pathogenesis of disease, the therapeutic strategies that targeted cGAS/STING/NLRP3 signaling pathway. We believe that targeting the cGAS/STING/NLRP3 signaling pathway in the future has broad prospects and significance for the treatment of disease.

## 2 cGAS/STING signaling pathway

**Figure 1:**
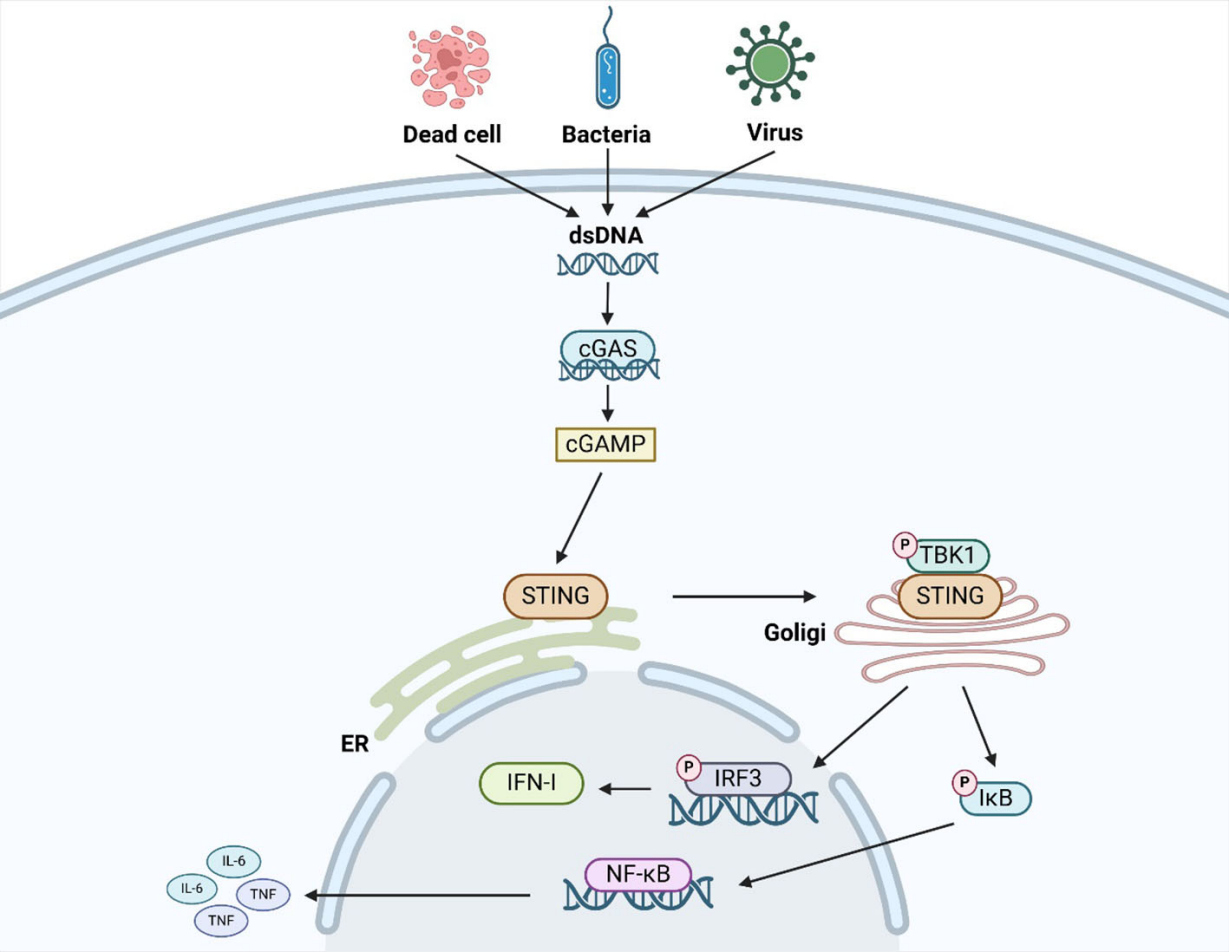
The mechanism of the canonical cGAS/STING signaling pathway.

More and more studies have found that the cGAS/STING signaling pathway plays an important role in infectious diseases, neurodegenerative diseases, autoimmune diseases, inflammatory diseases, liver diseases, cardiovascular diseases, and tumors (7, 8). Therefore, it is very important for us to understand the mechanism of the cGAS/STING signaling pathway (**Figure 1**). cGAS is a key component in the cGAS/STING signaling pathway. By recognizing and binding the dsDNA, cGAS converts adenosine triphosphate(ATP) and guanosine triphosphate(GTP) into 2 ‘-3’ ring GMP-AMP(cGAMP) (9). As a second messenger, cGAMP can directly bind to STING. After binding to cGAMP, STING dimer undergoes a transition from an inactive state to an active state and triggers its translocation from the endoplasmic reticulum to the Golgi apparatus (9). STING is phosphorylated by TBK1, leading to recruitment of IRF3. After being phosphorylated by TBK1, IRF3 enters the nucleus to drive type I interferon(IFN-I)expression. Meanwhile, STING also recruits IκB kinase (IKK), which phosphorylates iκBα (an inhibitor of NF-κB), resulting in the translocation of NF-κB to the nucleus and expression of pro-inflammatory cytokines (such as IL-6, TNF) (10). As mentioned above, The cGAS/STING/TBK1/IRF3 signaling pathway is the canonical cGAS/STING signaling pathway. This signaling pathway is characterized by the activation of IFN-I and inflammatory factors. Therefore, targeting this signaling pathway plays an important role in improving viral infections, tumors, autoimmune diseases, and inflammatory diseases. In addition to the canonical cGAS/STING signaling pathway, many noncanonical cGAS/STING signaling pathways have also been revealed in recent years. For example, Zhang D and his colleagues identified a noncanonical signaling pathway mediated by STING protein on the endoplasmic reticulum: the cGAS/STING/PERK/eIF2α signaling pathway (7). STING at the ER binds and directly activates the ER-located kinase PERK via their intracellular domains, which precedes TBK1–IRF3 activation and is irrelevant to the unfolded protein response. The activated PERK phosphorylates eIF2α, forming an inflammatory program. Inhibiting this signaling pathway can significantly improve cellular senescence, the pathological process of pulmonary fibrosis, and the pathological process of liver fibrosis. Liu D and his colleagues identified a noncanonical signaling pathway mediated by STING protein on endoplasmic reticulum Golgi intermediates (ERGIC): cGAS/STING/LC3-mediated autophagy (8). As a potential autophagy receptor, STING promotes the esterification reaction of LC3 and induces the occurrence of autophagy. Inhibiting this signaling pathway can significantly improve viral infections and inflammatory diseases. In short, the cGAS/STING signaling pathway is one of the most popular signaling pathways in recent years. It is of great significance to explore the mechanism and downstream pathway of this signaling pathway in these diseases.

## 3 NLRP3 inflammasome

The inflammasome is a multiprotein complex in the cytoplasm. Meanwhile, it is also a relatively important part of the immune system. The inflammasome includes NLRP1, NLRP2, NLRP3, NLRP6, NLRP12, NLRC4, AIM2. Among them, the NLRP3 inflammasome is the most studied and popular inflammasome (11). The NLRP3 inflammasome is composed of NLRP3, ASC, and pro-Caspase-1 (12). As a sensor for danger signals, NLRP3 inflammasome can be activated by pathogen-associated molecular patterns(PAMPs) and damage-associated molecular patterns(DAMPs) and then induce the maturation of pro-IL-1β and pro-IL-18 (12). In the physiological state, intracellular NLRP3 and downstream(pro-IL-1β and pro-IL-18) are at extremely low levels to maintain a low inflammatory state (13). When PAMPs or DAMPs are recognized by the corresponding PRRs, NF-κB nuclear translocation is triggered, and then the transcriptional expression of NLRP3, IL-1β, and IL-18 genes is activated (13). Oligomerization of the NLRP3 structural protein causes it to bind to the PYD of the splice protein ASC, and then the CARD of ASC binds to the CARD of pro-Caspase-1 to form an active NLRP3 inflammasome. The NLRP3 inflammasome promotes the self-cleavage of pro-Caspase-1 to produce active Caspase-1. Caspase-1 has the function of cutting GSDMD. On the one hand, Caspase-1 directly cleaved GSDMD to initiate pyroptosis (14). On the other hand, Caspase-1 can also induce the conversion of pro-IL-1β and pro-IL-18 to IL-1β and IL-18, which are secreted outside the cell to induce inflammation. This process is induced by a number of endogenous and exogenous agonists(ATP, cholesterol, silica, alum, amyloid). These agonists may activate the NLRP3 inflammasome through a number of common signaling pathways. At present, the activation pathways of NLRP3 inflammasome mainly include endoplasmic reticulum stress, mitochondrial damage, and lysosome damage (14). As an important substance in immune regulation, NLRP3 plays an important role in various diseases. Therefore, it is very interesting and meaningful for us to explore the relationship between the cGAS/STING signaling pathway and NLRP3.

## 4 cGAS/STING signaling pathway and NLRP3

**Figure 2:**
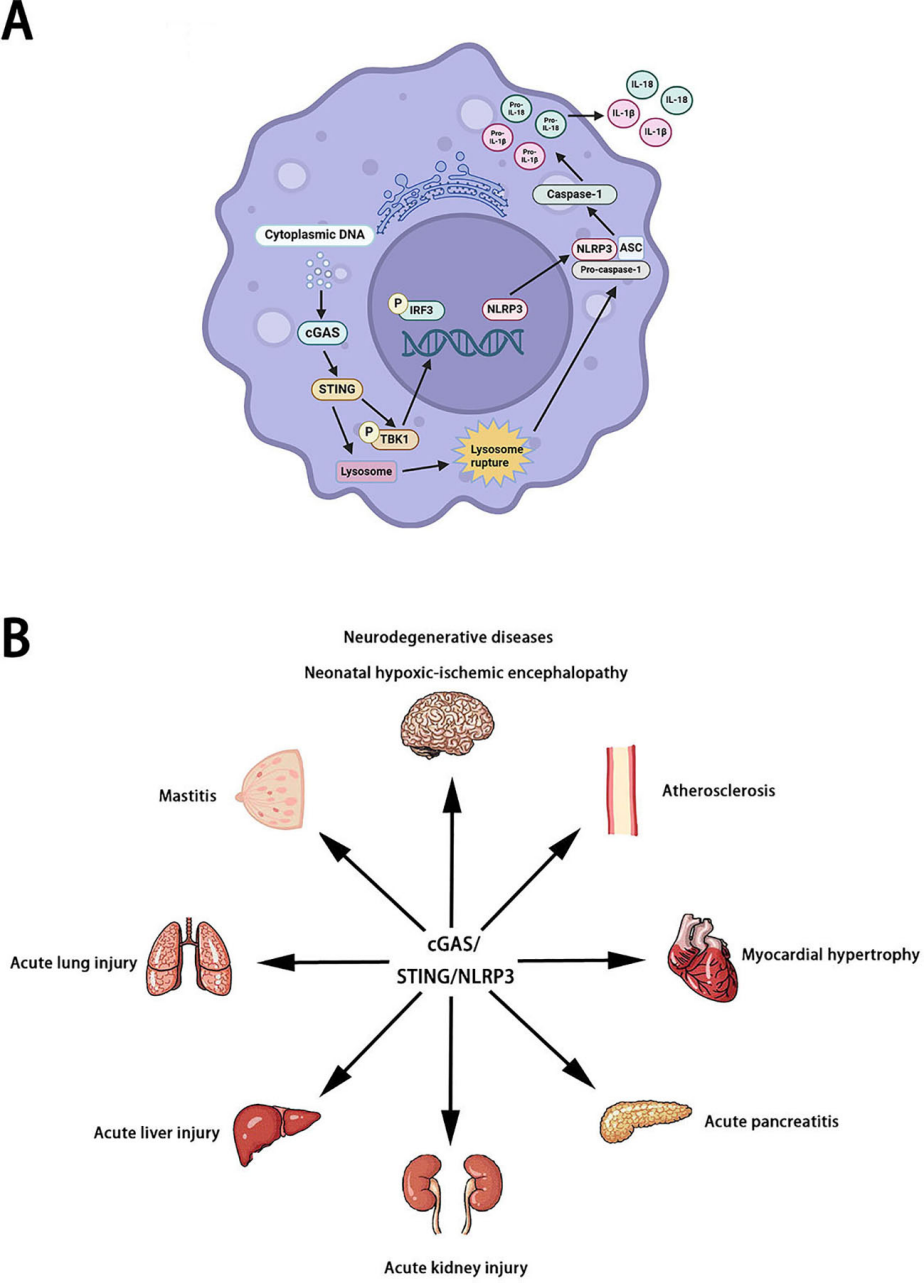
The pathogenesis **(A)** and role **(B)** of cGAS/STING/NLRP3 signaling pathway.

At present, more and more studies have proved that NLRP3 is the downstream of the cGAS/STING signaling pathway. NLRP3 is important for the cGAS/STING signaling pathway to perform various roles (15). NLRP3 deficiency increased the production of IFN-I (16). In neutrophils of sepsis mice, cGAS/STING signaling pathway can induce the activation of NLRP3 inflammasome to promote neutrophil pyroptosis (17). In myelodysplastic syndromes, cGAS/STING signaling pathway can activate the NLRP3 inflammasome by inducing ISG (18). STING/NLRP3 signaling pathway can also participate in lipopolysaccharide-induced inflammation and pyroptosis by activating NLRP3. In wild-type mice and cardiomyocytes, lipopolysaccharide stimulated the binding of STING to IRF3 and phosphorylate IRF3. Phosphorylated IRF3 is subsequently translocated into the nucleus and increases the expression of NLRP3 (19). Therefore, it is very interesting and meaningful to explore how the cGAS/STING signaling pathway acts on NLRP3. Available studies indicate that STING can promote the activation of NLRP3 inflammasome in a variety of ways under the stimulation of cytoplasmic DNA. On the one hand, STING activates NLRP3 inflammasome by recruiting NLRP3 and deubiquitinating NLRP3 (20). On the other hand, STING is activated and transported to lysosomes, triggering membrane penetration and leading to lysosomal cell death. After the death of lysozyme cells, the lysed cathepsin leaks into the cytoplasm, changes the permeability of the plasma membrane, activates K^+^ efflux(upstream of NLRP3), and finally induces pyroptosis (21, 22). In conclusion, NLRP3 is a potential downstream target for the cGAS/STING signaling pathway (**Figure 2**).

## 5 cGAS/STING/NLRP3 signaling pathway and diseases

Neurodegenerative diseases are a class of diseases characterized by the gradual death of neurons. According to the different pathogenesis, neurodegenerative diseases can be divided into Alzheimer’s disease, Parkinson’s disease, Huntington’s disease, and Amyotrophic lateral sclerosis. More and more studies have shown that the cGAS/STING/NLRP3 signaling pathway plays an important role in Neurodegenerative diseases. Harmful inflammatory environments contribute to the progression of neurodegenerative disorders. Serum/glucocorticoid related kinase 1 (SGK1), a Serine/Threonine protein kinase, is widely expressed in the heart, brain, liver, kidney, and other tissues. Previous studies have shown that SGK1 is involved in the pathogenesis of various neurodegenerative diseases. Kwon OC et al. found that SGK1 inhibition corrects the pro-inflammatory properties by regulating the cGAS/STING/NLRP3 signaling pathway (23). In sevoflurane-induced cognitive dysfunction mice, the cGAS/STING/NLRP3 signaling pathway was activated to induce neuroinflammation (24). SENP7 (SUMO specific peptidase 7) is a multifunctional enzyme. Liu L found that SENP7 induced neuronal apoptosis by regulating the cGAS/STING/NLRP3 signaling pathway in sevoflurane-induced cognitive dysfunction mice (25). Heavy metal pollution is a growing global problem that poses significant risks to the environment and human health. Copper exposure-induced neurotoxicity and manganese exposure-induced neurotoxicity may be associated with excessive activation of the cGAS/STING/NLRP3 signaling pathway (26, 27). Notably, cadmium exposure-induced pulmonary toxicity may be associated with excessive activation of the cGAS/STING/NLRP3 signaling pathway (28). Some other environmental pollutants, such as hexafluoropropylene oxide trimer acid and polystyrene micronanomaterials, can also induce liver fibrosis and spermatogenesis dysfunction in mice by regulating the cGAS/STING/NLRP3 signaling pathway (29, 30). In addition to neurodegenerative diseases, inhibition of the cGAS/STING/NLRP3 signaling pathway can also improve neonatal hypoxic-ischemic encephalopathy and cerebral venous sinus thrombosis (31, 32). Cardiovascular disease (CVD) is a disease involving the heart or blood vessels, including atherosclerosis, heart failure, myocardial infarction, cardiomyopathy, arrhythmia, and coronary heart disease. In diabetic cardiomyopathy mice, free fatty acids promoted myocardial hypertrophy by activating cGAS/STING/NLRP3 signaling pathway (33). In myocardial infarction patients, high levels of dsDNA directly potentiate platelet activation via the cGAS/STING/NLRP3 signaling pathway (34). IQ motif-containing GTPase-activating protein1 (IQGAP1) is an important scaffolding protein. An C et al. found that IQGAP1 induces endothelial cell pyroptosis leading to atherosclerosis via the cGAS/STING/NLRP3 signaling pathway (35). In addition to neurodegenerative diseases and cardiovascular diseases, the cGAS/STING/NLRP3 signaling pathway is involved in the pathological process of acute pancreatitis (36), mastitis (37), acute kidney injury (38), acute liver injury (39), acute lung injury (40), systemic lupus erythematosus (41), and allergic rhinitis (42). Unfortunately, only a few studies have explored the relationship between the cGAS/STING/NLRP3 signaling pathway and disease. Therefore, it is very promising to explore the mechanism and relationship of the cGAS/STING/NLRP3 signaling pathway in various diseases in the future.

## 6 cGAS/STING/NLRP3 signaling pathway and therapeutic strategies

Because the cGAS/STING/NLRP3 signaling pathway plays an important role in the pathogenesis of various diseases, more and more studies are beginning to explore therapeutic strategies that target the cGAS/STING/NLRP3 signaling pathway. As we all know, natural products have the advantages of good efficacy, low toxicity, low cost, wide source, and high yield in the treatment of diseases. Gao D et al. found that tetrahydroxy stilbene glucoside inhibited neuroinflammation through the cGAS/STING/NLRP3 signaling pathway (43). Lai Y et al. found that Dihydrocapsaicin promoted the perforator flap survival by suppressing the cGAS/STING/NLRP3 signaling pathway (44). Tian Y et al. found that epigallocatechin gallate exerts anti-inflammatory activity by inhibiting the cGAS/STING/NLRP3 signaling pathway (45). Saikosaponin D is an active compound derived from the Radix bupleuri. In cerulein-induced pancreatic acinar cells (PACs) injury mode, Saikosaponin d protects PACs against pyroptosis by inhibiting the cGAS/STING/NLRP3 signaling pathway (46). Emodin is an anthraquinone derivative that is present in numerous herbal medicines. In acetaminophen-induced C57BL/6 mice, emodin protects hepatocytes from liver injury by inhibiting cGAS/STING/NLRP3 signaling pathway (47). Chlorogenic acid(CA), also known as 5-O-caffeoylquinic acid, is a phenolic compound. In varicocele rats, CA can improve spermatogenic dysfunction by inhibiting the cGAS/STING/NLRP3 signaling pathway (48). In addition to natural products, some chemical agents such as melatonin, Pulmozyme, and C-176 can also improve disease by regulating the cGAS/STING/NLRP3 signaling pathway (49–51). Interestingly, some non-drug treatment strategies have also been reported. For example, electroacupuncture alleviates depression in mice by regulating the cGAS/STING/NLRP3 signaling pathway (52). Transplanting fecal microbiota mitigated neurotoxicity by regulating the cGAS/STING/NLRP3 signaling pathway (53). Hydrogen sulfide improves cardiac dysfunction by inhibiting the cGAS/STING/NLRP3 signaling pathway (54). Together, these therapeutic strategies have important implications for improving and safeguarding human health. More treatment strategies need to be explored in the future.

## 7 Discussion

**Figure 3:**
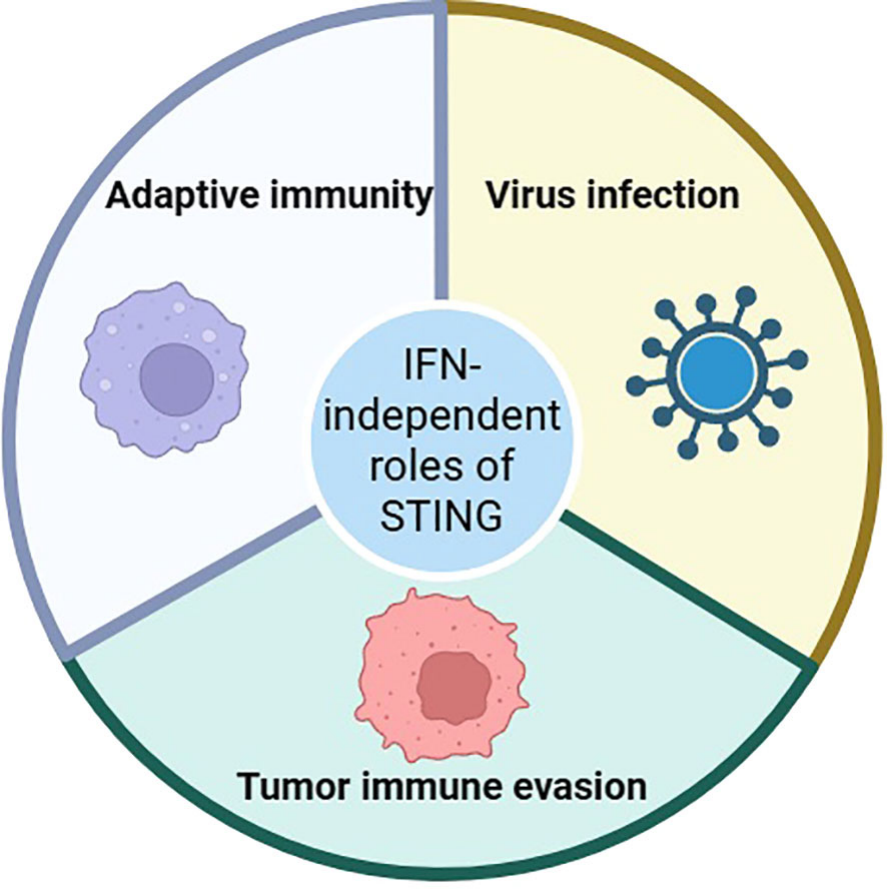
The IFN-independent roles of STING.

In recent years, as a hot signaling pathway, the cGAS/STING signaling pathway play an important role in bacterial infection, viral infection, immune diseases, and inflammatory diseases. On the one hand, the cGAS/STING signaling pathway induces the production of IFN-I by activating the TBK1 and IRF3. On the other hand, the cGAS/STING signaling pathway allows NF-κB to enter the nucleus and induce inflammatory responses by recruiting the IKK. At present, many noncanonical cGAS/STING signaling pathways have also been revealed. Studying IFN-independent roles of STING is challenging due to the overwhelming effects of IFN signaling when STING is activated by agonists, and IFN may mask other activities of STING. Interestingly, many IFN-independent roles of cGAS or STING have been reported to date. For example, Wu J et al. found that STING controls herpes simplex virus-1 (HSV-1) infection through IFN-independent activities (55). Wang K et al. found that STING controls hantaviral (HTNV) infection through IFN-independent way (56). Another important finding is that STING activates many signaling pathways in T cells and the majority of them are IFN-independent (57). These data demonstrate that mammalian STING possesses widespread IFN-independent activities that are important for restricting virus infection, tumor immune evasion, and adaptive immunity (**Figure 3**). It is very important to explore the connection between STING and NLRP3. The recent evidence indicates that stimulating STING prompts the activation of the NLRP3 inflammasome. STING comprises five putative transmembrane (TM) regions. TM5 (151-160 aa, human) is involved in the interaction with NLRP3, while TM2 (41-81 aa, human) participates in the assembly and activation of the NLRP3 inflammasome (58). STING binds NLRP3, and then it can promote the activation of the inflammasome via NLRP3 localization and the removal of NLRP3 polyubiquitination (59). Therefore, we believe that NLRP3 is a better downstream target for STING. As a downstream of the cGAS/STING signaling pathway, NLRP3 has been demonstrated in the pathogenesis of an increasing number of diseases. Many natural products, chemical agents, and non-drug treatment strategies can regulate this signaling pathway to improve disease. It is worth noting that some studies have also pointed out that the NLRP3 inflammasome and its downstream Caspase-1, GSDMD can also regulate the cGAS/STING signaling pathway. For example, Wang Y and his team found that Caspase-1 cleaved cGAS and inhibited STING-mediated IFN production during inflammasome activation (60). Banerjee I and his team found that GSDMD negatively regulates IFN in a manner independent of pyroptosis and IL-1β (61). These studies indicate the presence of crosstalk networks in the cGAS/STING signaling pathway and NLRP3. Therefore, more studies are needed in the future to explore the association between the cGAS/STING signaling pathway and NLRP3.
